# Supplementary material for: Pharmacological inhibition of α-synuclein aggregation within liquid condensates
Source: Nat Commun. 2024 May 7;15:3835. doi: 10.1038/s41467-024-47585-x (PMC11076612; doi:10.1038/s41467-024-47585-x)
Supplement: Supplementary file 1 — Supplementary Information [file 41467_2024_47585_MOESM1_ESM.pdf]

Supplementary Information

Pharmacological inhibition of α-synuclein aggregation within liquid condensates

Samuel T. Dada, Zenon Toprakcioglu, Mariana P. Cali, Alexander Röntgen, Maarten C. Hardenberg, Owen M. Morris, Lena K. Mrugalla, Tuomas P.J. Knowles and Michele Vendruscolo*

Centre for Misfolding Diseases, Department of Chemistry, University of Cambridge, Cambridge CB2 1EW, UK

Table S1. Rate constants of the microscopic processes involved in α-synuclein aggregation within liquid condensates. We used 75 µM α-synuclein in the absence (1% DMSO) and presence of claramine at 25, 50 and 75 µM. The rates obtained from kinetic traces are displayed in **Supplementary Figure 8**).

<table>
<tr><th>Conditions</th><th>Primary nucleation
K_n
($M^{-1}min^{-1}$)</th><th>Secondary nucleation
K_2
($M^{-2}min^{-1}$)</th><th>Elongation
K_+
($M^{-1}min^{-1}$)</th></tr>
<tr><td>Control</td><td>0.825</td><td rowspan="4">1.97×10^7</td><td rowspan="4">8.73×10^4</td></tr>
<tr><td>25 µM Claramine</td><td>0.0201</td></tr>
<tr><td>50 µM Claramine</td><td>0.00603</td></tr>
<tr><td>75 µM Claramine</td><td>0.0000351</td></tr>
</table>

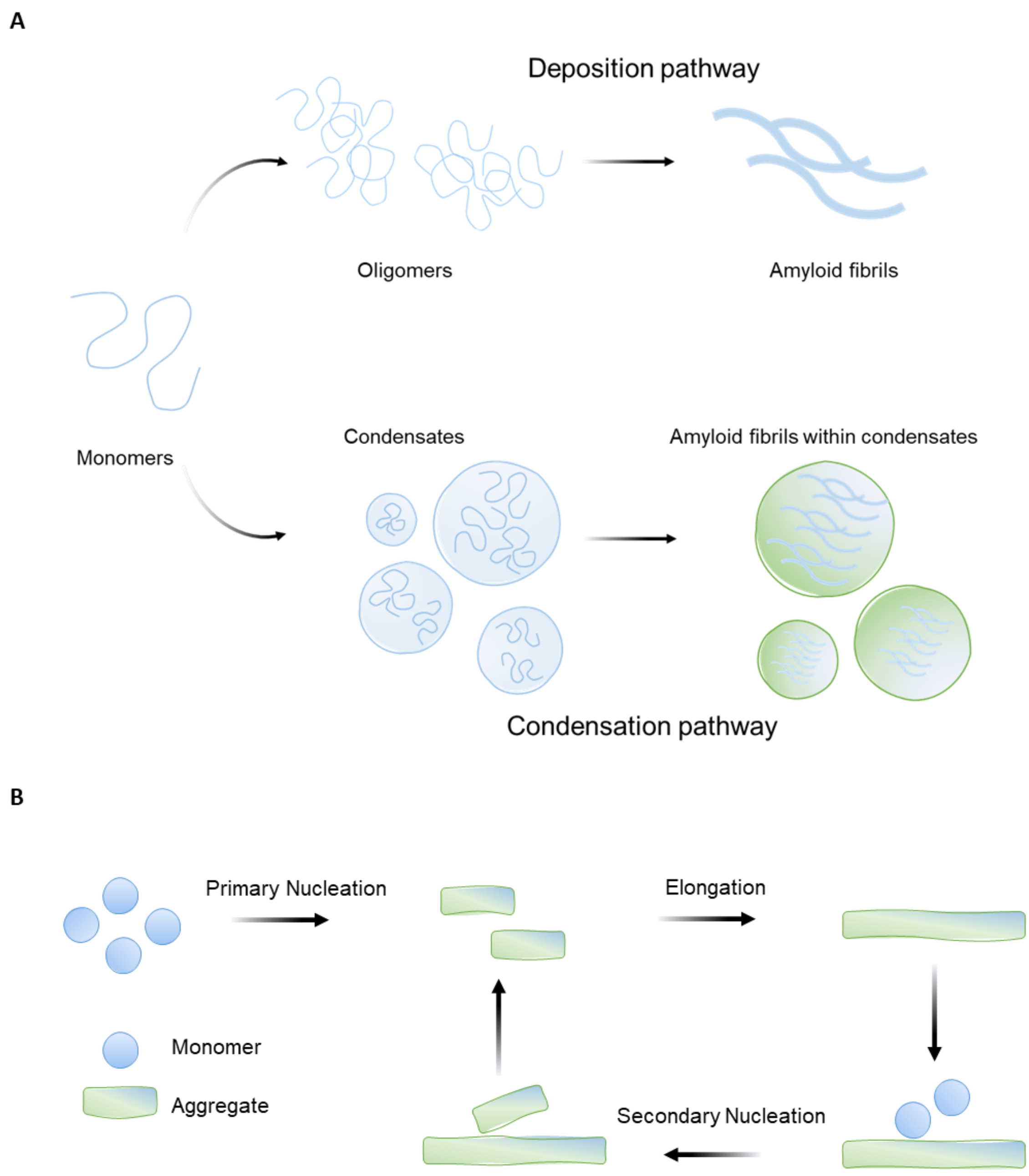

Supplementary Figure 1. Comparison of the deposition and condensation pathways of α-synuclein aggregation. (A) The conversion of α-synuclein from the monomeric to the amyloid states can occur through two distinct pathways[1-6]. In the deposition pathway, α-synuclein initially forms oligomers that then grow into amyloid fibrils. In the condensation pathway, α-synuclein first forms condensates, where the high concentration promotes the nucleation of initial aggregates, which then grow into amyloid fibrils. **(B)** A kinetic network that models the proliferation of α-synuclein aggregates after the initial formation of amyloid fibrils shown in panel A. This kinetic network is conserved in the deposition and condensation pathways[7]. The process begins with a primary nucleation step, where monomers spontaneously assemble into amyloid fibril seeds. Subsequently, these seeds grow through elongation into mature amyloid fibrils. The presence of existing filaments catalyses the formation and nucleation of new aggregates on the surface.

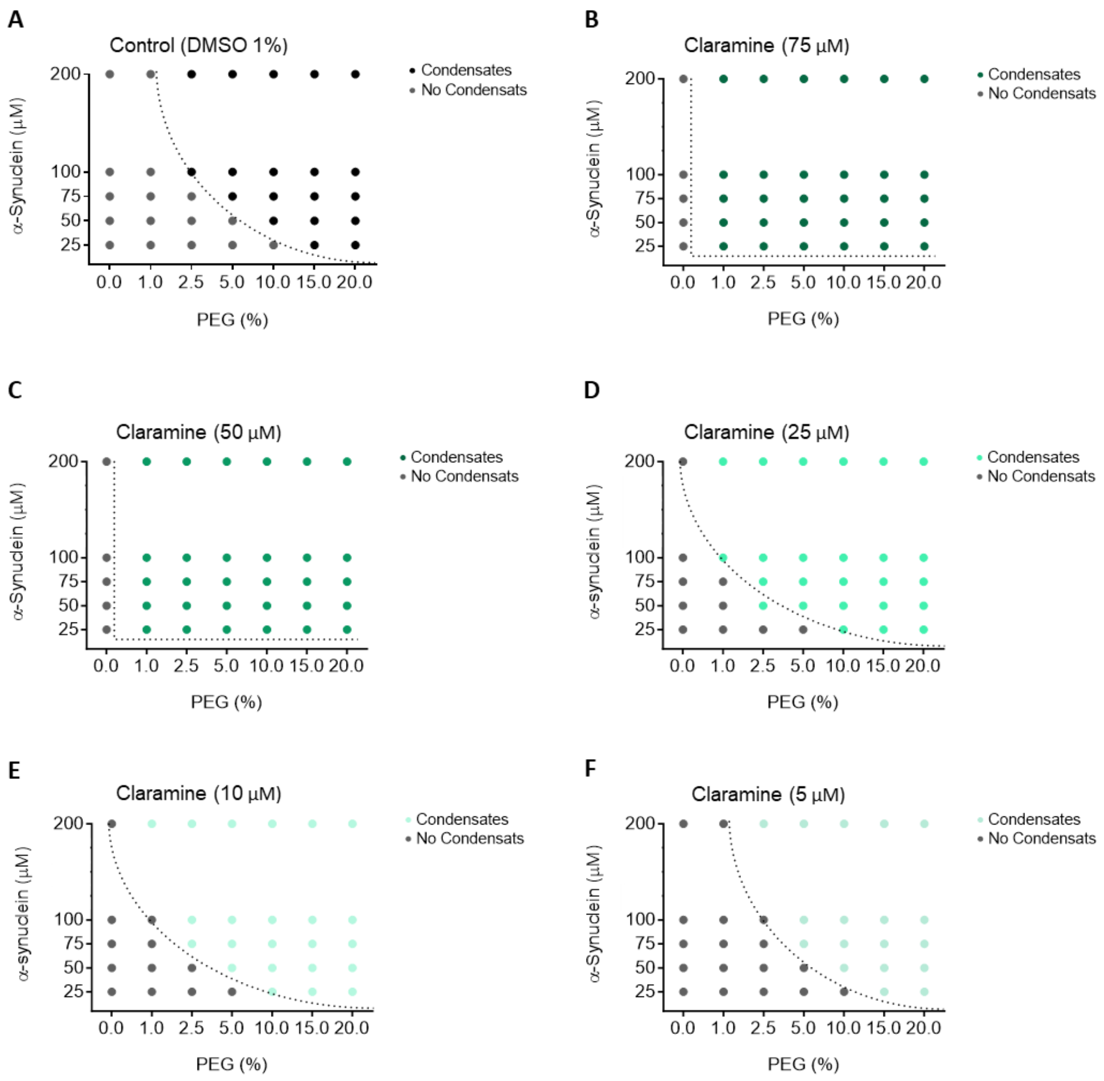

Supplementary Figure 2. Claramine shifts the phase boundary of α-synuclein. (A-E) Phase diagram for different PEG and α-synuclein concentrations (1% DMSO) in the absence **(A)** and presence of claramine at concentrations of 75 µM **(B)**, 50 µM **(C)**, 25 µM **(D)**, 10 µM **(E)** and 5 µM **(F)** at which phase separation was observed after a 10 min incubation period. The dots indicate the tested conditions, the grey dots for lack of phase separation, and the colored dots for condensates. The dotted black lines represent the phase boundary. All experiments were performed in 50 mM Tris-HCl at pH 7.4. The data shown are representative of experiments repeated at least three times.

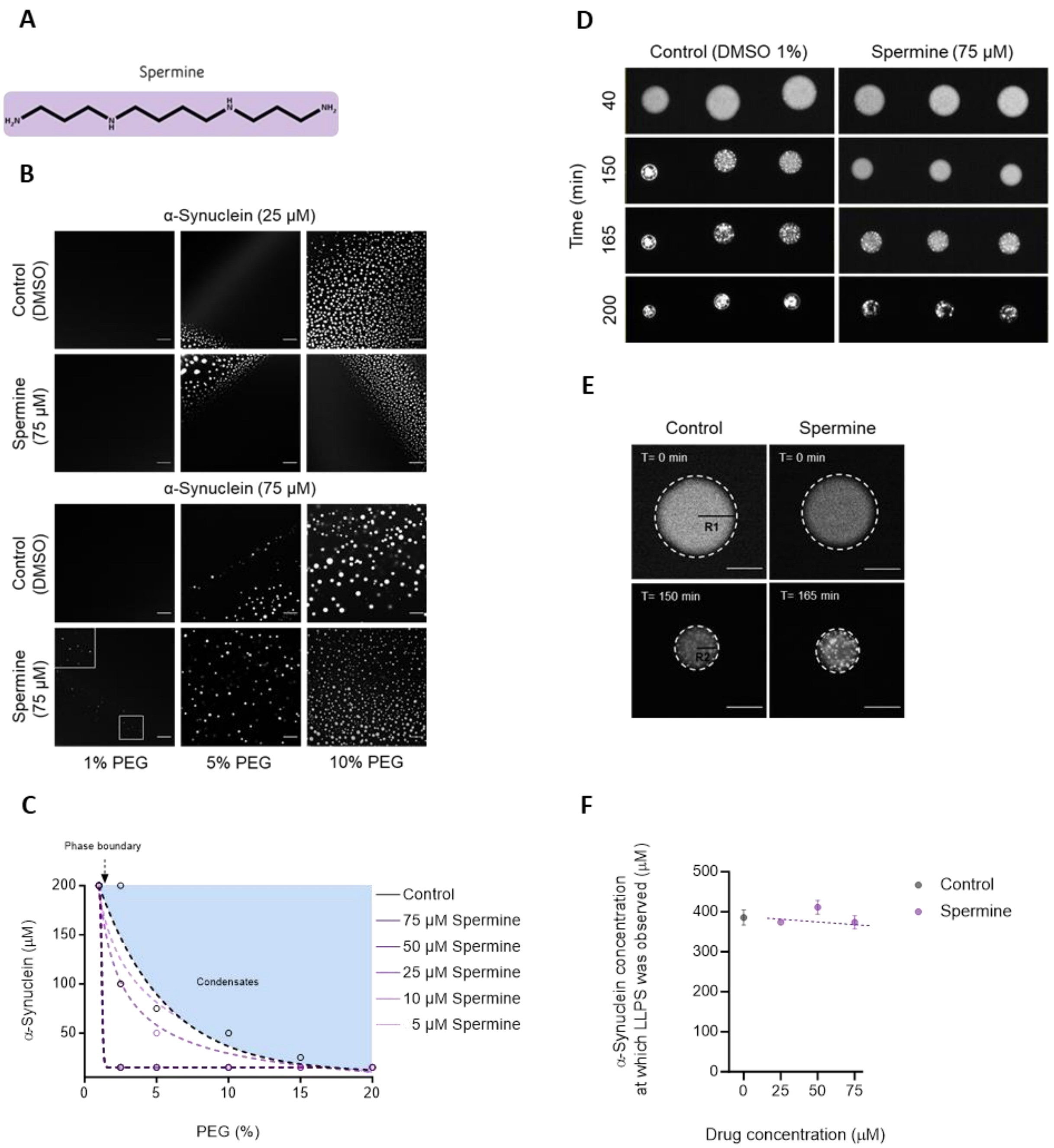

Supplementary Figure 3. Spermine does not change the propensity of α-synuclein to undergo phase separation. (A) Chemical structure of spermine, a polyamine bearing four amino groups. **(B)** Representative fluorescence images of α-synuclein condensate formation in the presence and absence (DMSO 1%) of spermine (75 µM) at different PEG concentrations. Images were visualised using Alexa Fluor 647 and obtained 10 min post incubation. Scale bar represents 20 µm. **(C)** Summary phase diagram showing the relationship between different α-synuclein and PEG concentrations the absence (control) and presence of spermine of different concentrations (75, 50, 25, 10, and 5 µM) at which phase separation was observed after a 10 min incubation period. The phase boundary, based on experimental observations for the different conditions tested, is represented by the dotted line; the increasing concentration of spermine is highlighted by the purple increasing gradient color. **(D-F)** α-Synuclein

concentration required for phase separation in the presence and absence of spermine, which was obtained using a microfluidic device (see Methods). **(D)** Representative fluorescence images displaying condensate formation of six droplets (first three droplets (no spermine) last three droplets (spermine 75 µM)) trapped within a microfluidic chamber overtime (40, 150, 165 and 200 min). **(E)** Enlarged images showing droplet in the absence and presence of spermine (75 µM) at the beginning of the experiment (0 min) and at the time of condensate formation (150 and 165 min for DMSO (1%) and spermine (75 µM) respectively). The scale bar represents 50 µm. **(F)** Concentration of α-synuclein at which phase separation was observed within droplets as shown in panels D and E, in the absence (grey) and presence of different concentrations of spermine (25, 50 and 75 µM (purple)) in the presence of 10% PEG. The concentration was calculated to be an average of 386, 374, 411, and 374 µM for DMSO (1%), 25, 50 and 75 µM spermine respectively. All experiments were performed in 50 mM Tris-HCl at pH 7.4 in the presence of 10% PEG unless otherwise stated. The data shown are representative of experiments repeated at least three times. Results are shown as mean ± SEM.

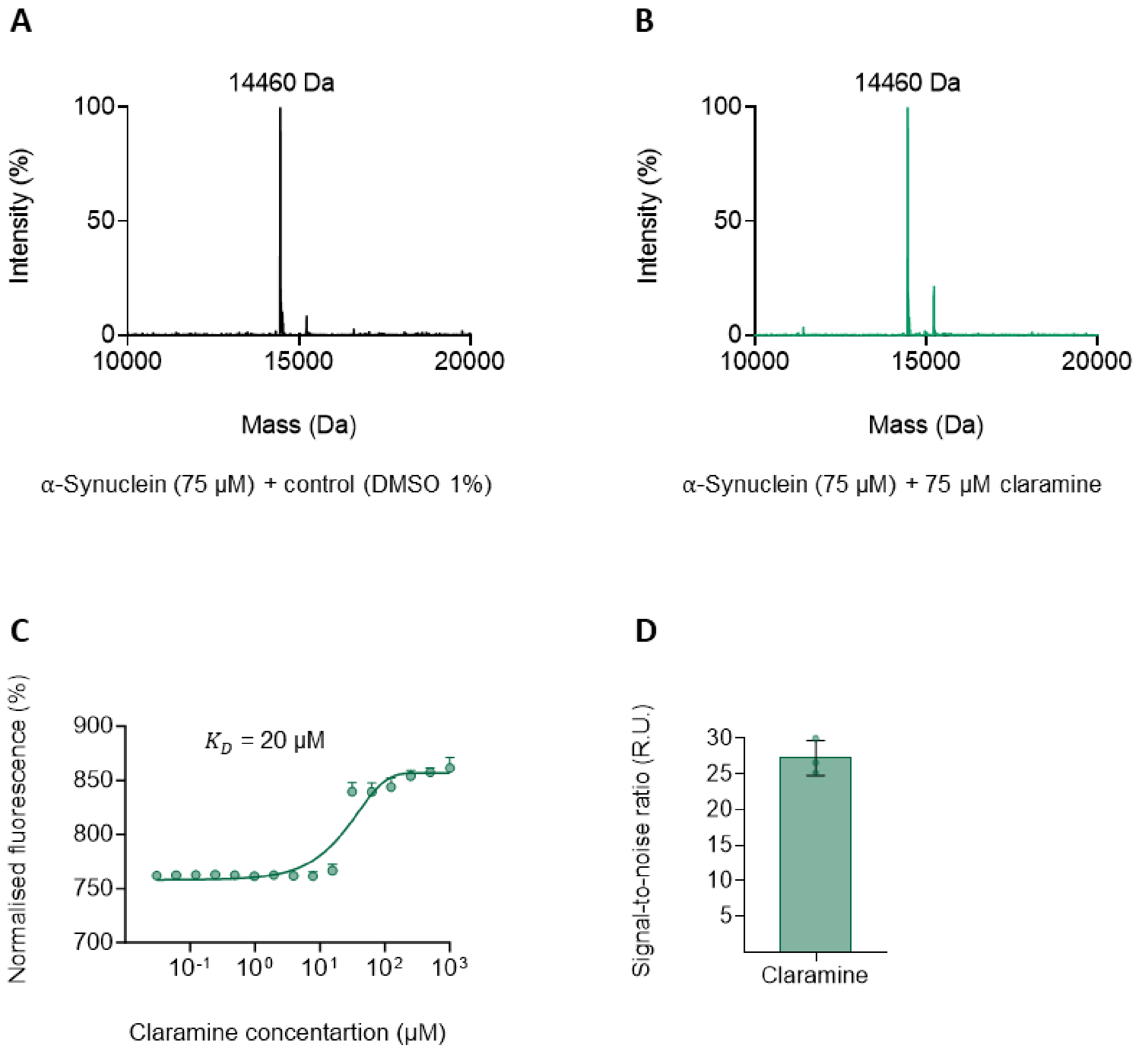

Supplementary Figure 4. Claramine does not covalently bind to α-synuclein. (A,B) LC-MS spectra of α-synuclein in the absence **(A)** and presence of claramine **(B)**. The observed and calculated mass of 14,460 Da is a confirmation of the presence of α-synuclein. All experiments were performed using 75 μM α-synuclein in 50 mM Tris-HCl at pH 7.4 in the presence of 10% PEG. **(C)** Thermophoretic measurements binding curve for claramine to α-synuclein (KD 20 μM). **(D)** Signal-to-noise ratio as a measure of the quality of the obtained binding data. For thermophoretic experiments α-synuclein concentration was kept constant at 1.5 μM with a 2% subset of monomers being labelled with Alexa Fluor 647. Measurements were performed at 22 °C with a 30% red LED intensity and a 50% IR laser intensity (laser being on for 30 sec).

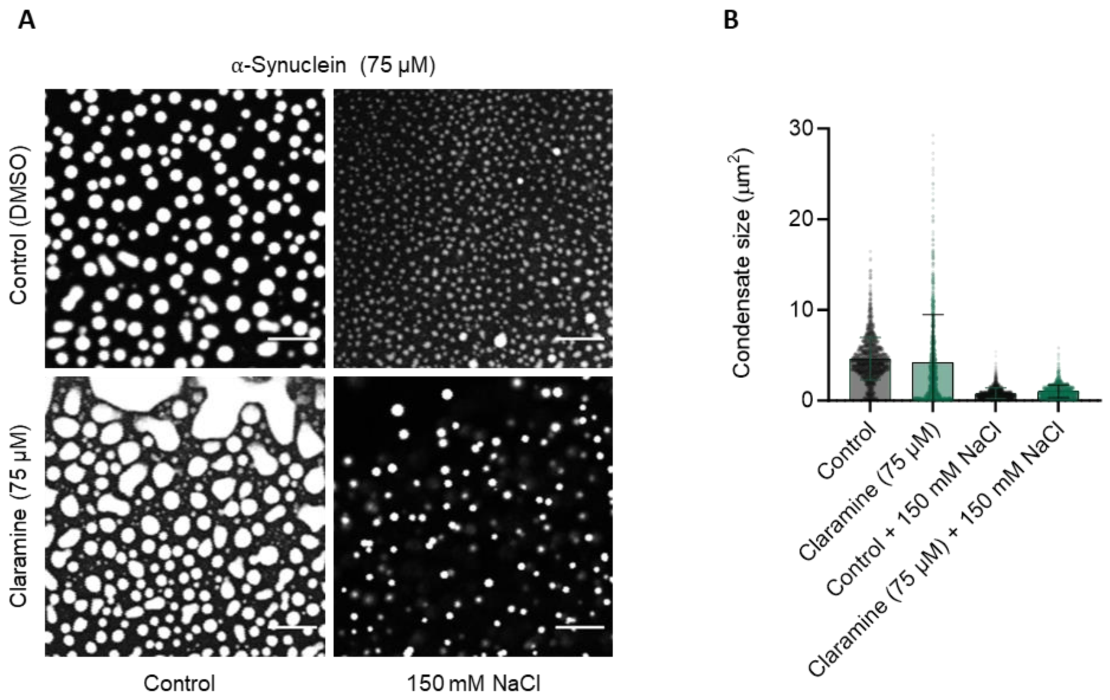

Supplementary Figure 5. High ionic strength alters the phase separation behaviour of α-synuclein in the presence and absence of claramine. (A) Fluorescence images of α-synuclein (75 µM) condensate formation in the presence and absence (1%DMSO) of claramine (75 µM) and 150 mM NaCl. Images were visualised using Alexa Fluor 647 and obtained at the 10 min time point post phase separation. Scale bar represents 10 µm. **(B)** Size distribution of representative images shown in panel A representing the quantification of the effect of NaCl (150 mM) in the buffer solution on the size of α-synuclein condensates at the 10 min time point. All experiments were performed in 50 mM Tris-HCl at pH 7.4 in the presence of 10% PEG unless otherwise stated. Results are shown as mean ± SD.

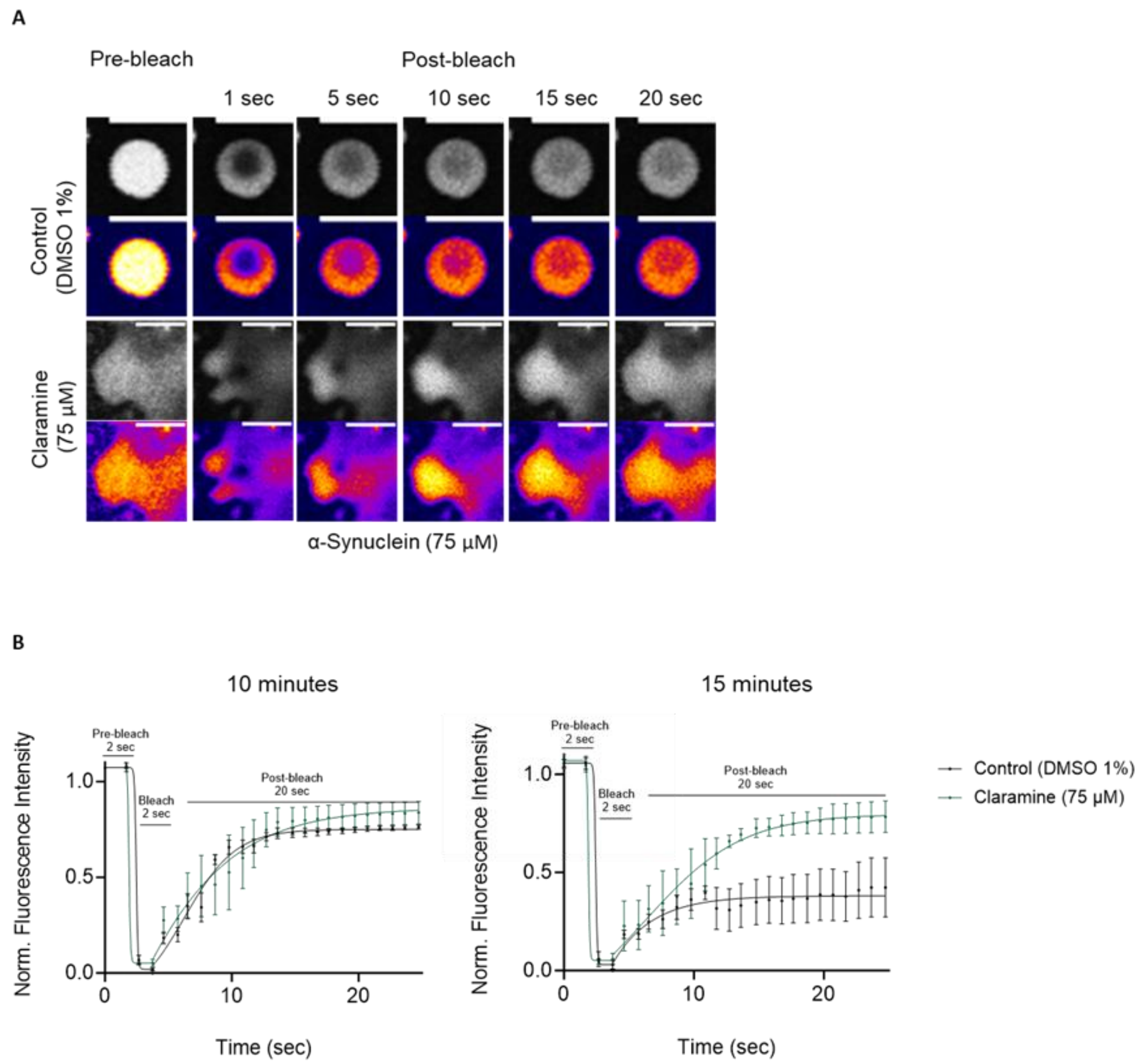

Supplementary Figure 6. Claramine enhances the fluidity of α-synuclein condensates. FRAP measurements of 75 µM α-synuclein condensates at 10 minutes and 15 minutes post onset of phase separation in the presence and absence of claramine (75 µM) to assess changes in condensate dynamics over time. **(A)** The representative images illustrate the region of interest before bleaching (2 sec) and after bleaching at 1, 5, 10, 15, and 20 seconds for each condition at the 15-minute time point post-onset of phase separation. Scale bar: 5 µm. **(B)** Normalised recovery traces from FRAP experiment for α-synuclein condensates at the 10 and 15-minute timepoint in the presence and absence of claramine. Data are shown as mean ± SEM of 4 technical and 4 biological replicates (n=4). Experiments were conducted in 50 mM Tris-HCl at pH 7.4 with 10% PEG.

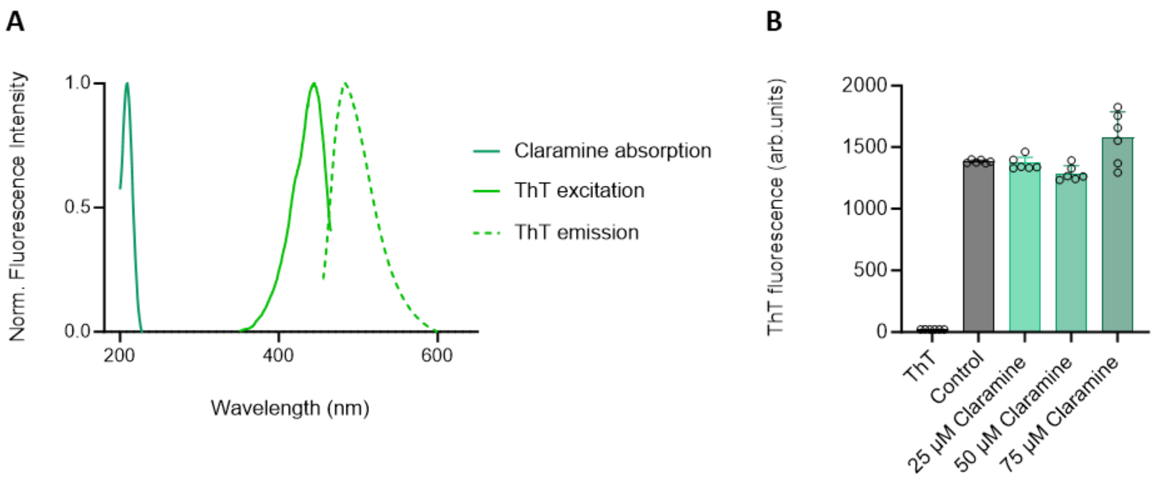

Supplementary Figure 7. Claramine does not quench the ThT signal. (A) Absorption spectra of claramine (209 nm) and excitation and emission spectra of ThT (excitation 440 nm, emission 480 nm) in the presence of 50 Mm Tris-HCL pH 7.4. **(B)** ThT fluorescence intensity of solution in the presence and absence (1% DMSO (black) of different concentrations of claramine (25 µM (light green), 50 µM (green) and 75 µM (dark green)). No fluorescence quenching is observed for any of the concentrations tested, as there was no significant decrease in ThT signal intensity in the presence of the molecule relative to the control regardless of claramine concentration.

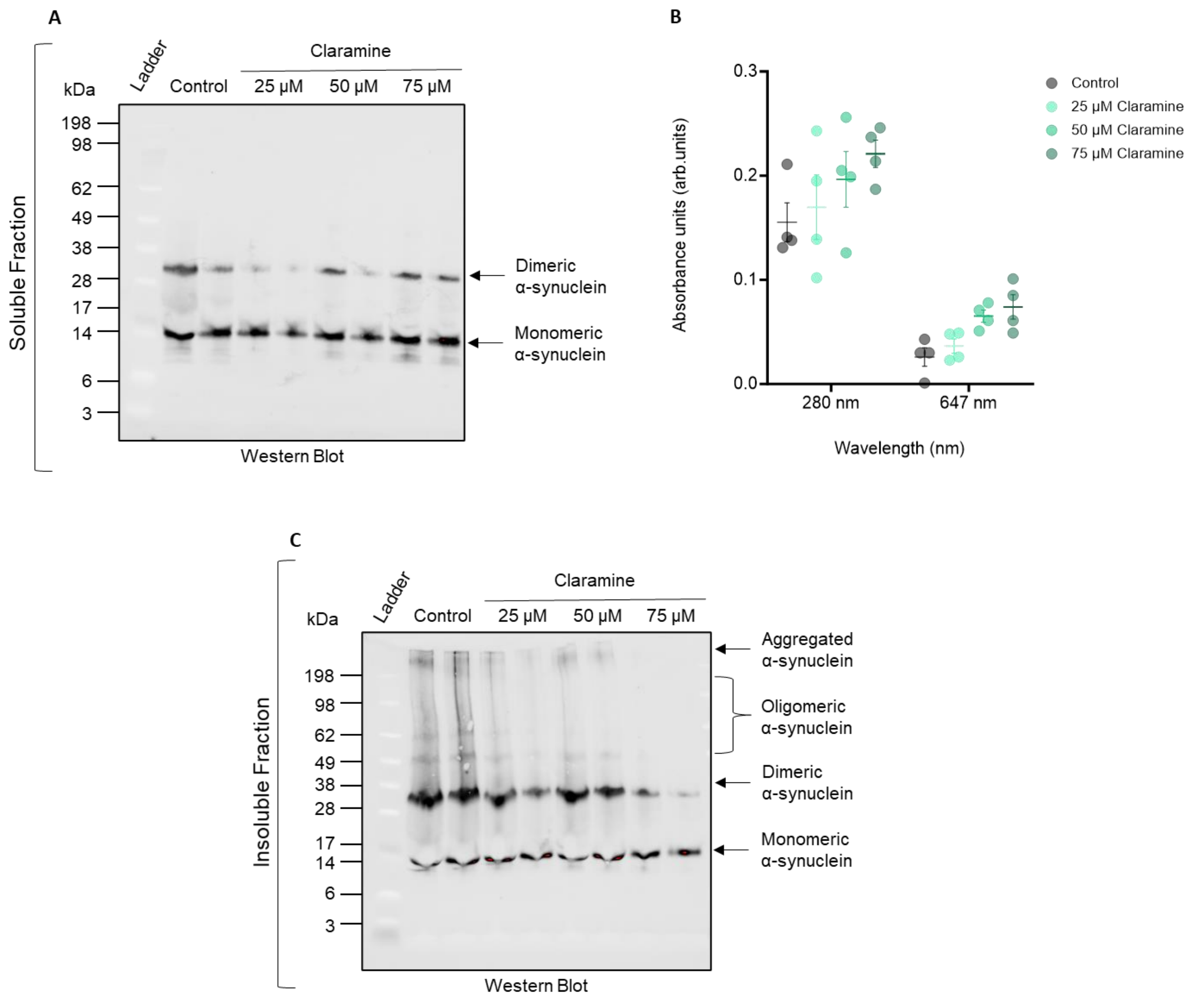

Supplementary Figure 8. Biochemical analysis of the effects of claramine on α-synuclein aggregation within condensates. (A-B) Analysis of soluble α-synuclein samples (found in sample supernatant) extracted from phase separation assay in the absence (DMSO) and presence of claramine (25, 50 and 75 µM). **(A)** Western blot analysis displaying the extracted soluble fraction in the presence and absence of claramine in duplicates. The western blots show the presence of a high concentration of α-synuclein monomer and a small amount of α-synuclein dimers at ~14 kDa and ~36 kDa, respectively, detected with the MJFR1 antibody. **(B)** Absorption at 280 nm (α-synuclein) and 647 nm (labelled α-synuclein Alexa Fluor 647) of soluble α-synuclein fractions in the absence (DMSO) and presence of claramine (25, 50 and 75 µM). **(C)** Analysis of insoluble α-synuclein samples (found in sample pellet) extracted from phase separation assay in the absence (DMSO) and presence of claramine (25, 50 and 75 µM) in duplicates. Western blot analysis confirming the detection of α-synuclein monomers, dimers, oligomers, and aggregates at ~14 kDa, ~36 kDa, between ~64 to 148 kDa and ~200 kDa respectively with MJFR1 antibody. All experiments were performed in 50 mM Tris-HCl at pH 7.4 in the presence of 10% PEG unless otherwise stated. The data shown are representative of experiments repeated at least three times. Results are shown as mean ± SEM.

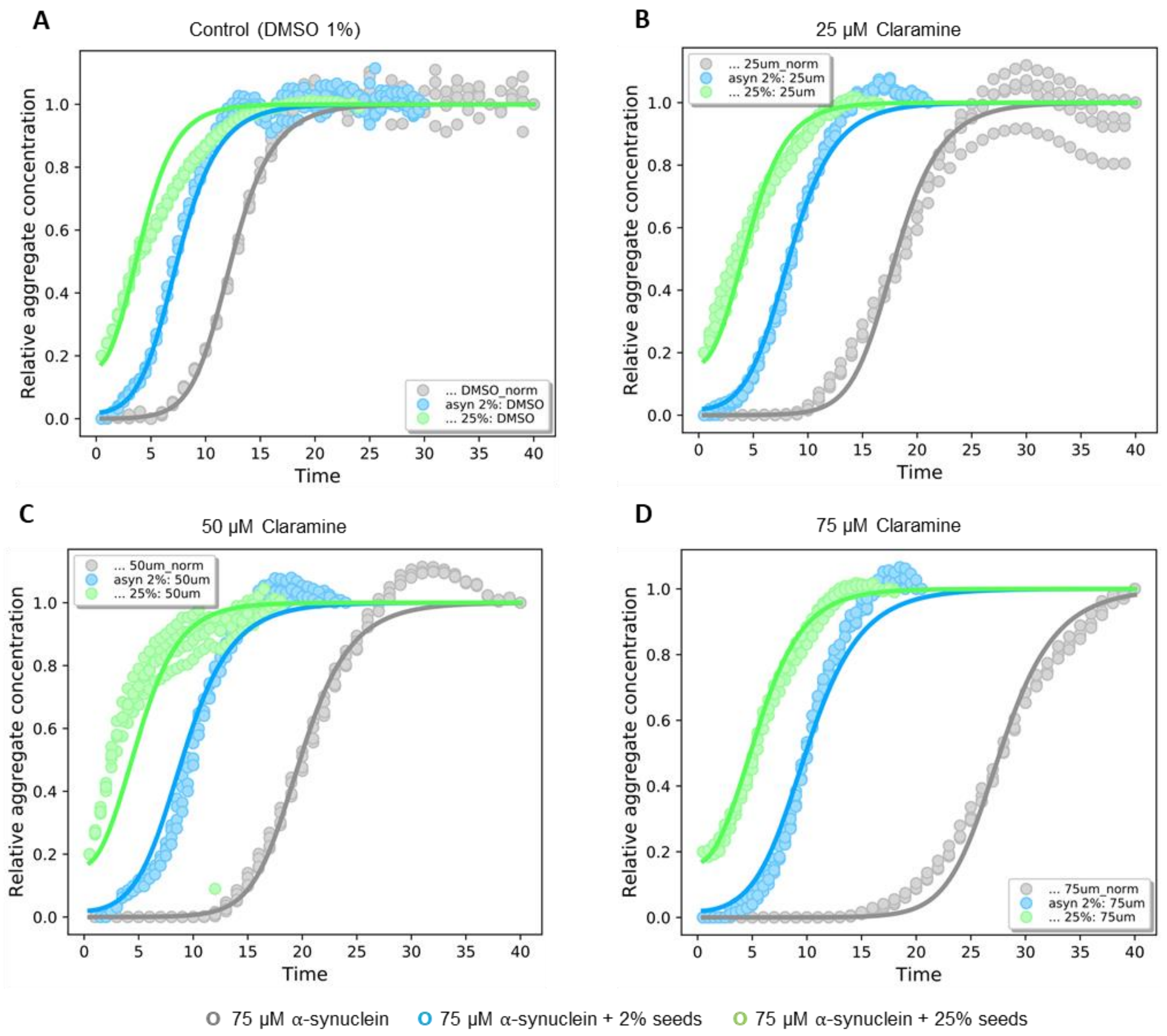

Supplementary Figure 9. The fit of the α-synuclein aggregation kinetics within liquid condensates in the presence of claramine indicates an increase in the primary nucleation rate. Global analysis of the kinetic traces to quantify the effect of claramine on α-synuclein aggregation within the liquid condensates. **(A-D)** traces of α-synuclein (75 µM (grey)) with 2% (blue) and 25% (green) seeds in the absence (control (1% DMSO) **(A)** and presence of claramine at 25 µM **(B)**, 50 µM **(C)** and 75 µM **(D)**. All experiments were performed using 75 µM α-synuclein with either 2% or 25% preformed fibrils in 50 mM Tris-HCl at pH 7.4 in the presence of 10% PEG unless otherwise stated. The data are shown as the mean ± SEM of n=4 individual experiments.

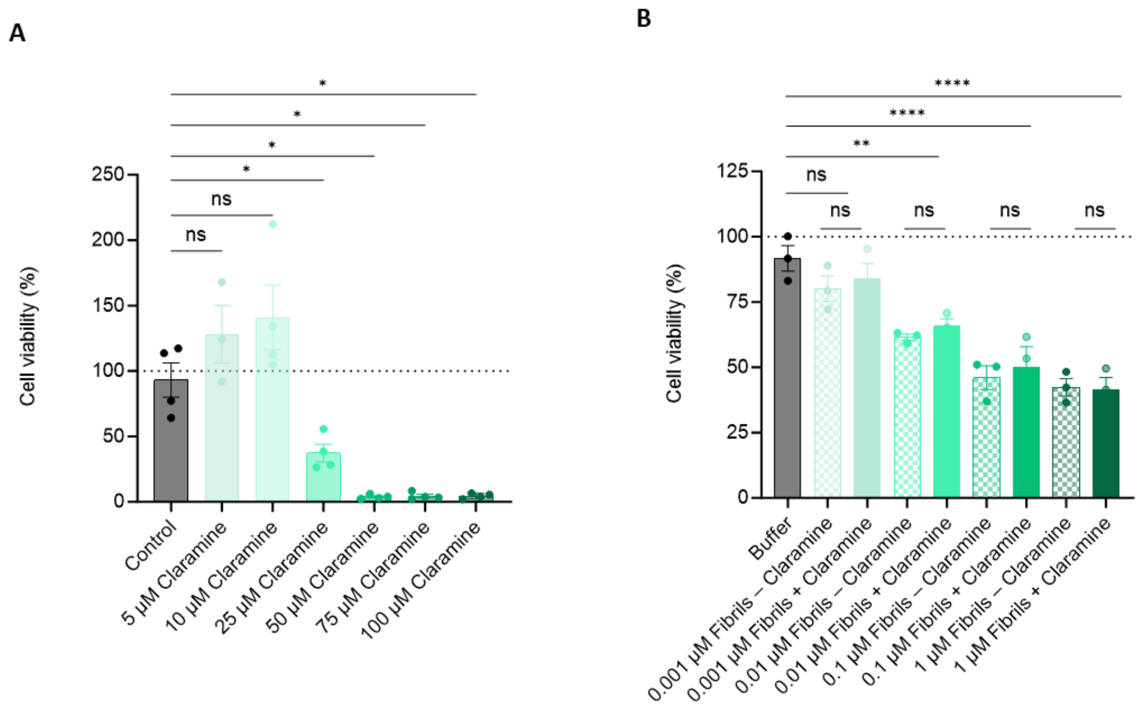

Supplementary Figure 10. Claramine does not suppress the toxicity of α-synuclein fibrils in human neuroblastoma SH-SY5Y cells. (A) Impact of claramine on SH-SY5Y cell viability after 24 h of treatment. Cells were treated with DMSO (grey), and varying concentrations of claramine (5, 10, 25, 50, 75, and 100 μM; green bars). **(B)** Effect of α-synuclein fibrils on SH-SY5Y cell viability after 24 h of treatment. The experiments were conducted both in the absence (buffer condition, represented by grey bars) and presence of α-synuclein fibrils at various concentrations (monomer equivalent; 0.001, 0.01, 0.1, and 1 μM, indicated by green bars) after 24 h of incubation. These fibrils were generated from aggregation assays, and the impact of claramine treatment at an equal molar ratio concentration to monomeric α-synuclein was examined. Light bars denote conditions without claramine, while dark bars, maintained at equivalent concentrations, represent the presence of claramine. Comparisons were carried out by a one-way ANOVA test with Dunnett's multiple comparisons correction for panel **A** and by a two-way ANOVA test with Šidák's multiple comparisons correction for panel **B** (n.s - not significant, *P < 0.1, **P < 0.01 ***P < 0.001, ****P < 0.0001). All bars indicate mean ± SEM of at least n = 3 biologically independent experiments (dots).

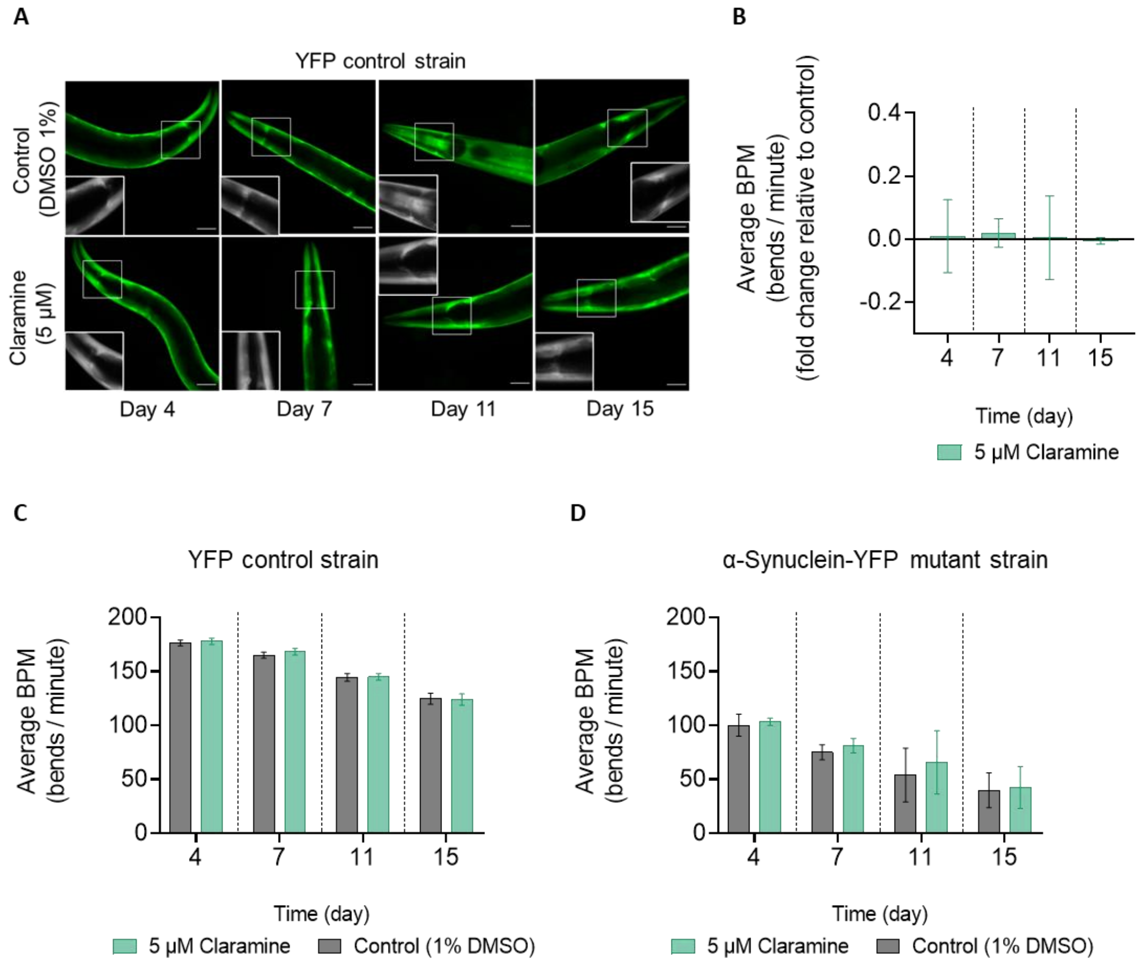

Supplementary Figure 11. Claramine administration did not lead to observable negative effects in YFP control *C. elegans*. **(A)** Microscopy images showing the effect of the administration of 1% DMSO or claramine (5 µM) to the YFP expression in the body wall muscle cells in the YFP control strain over time between days 4 and 15 of adulthood. The scale bar represents 20 µm. **(B)** Data from automated worm motility assay showing the relative fold change in average bends per minute of worms treated with 5 µM claramine over time between days 4 and 15 of adulthood in YFP control strain. **(C-D)** Raw data from automated nematode platform showing the average bends per minute for untreated nematodes and claramine-treated nematodes over time for YFP control strain **(C)** and α-synuclein-YFP mutant strain **(D)**. At least fifty worms were analysed in total per experiment. The data are shown as the mean ± SEM of at least 3 individual experiments.

Supplementary References

1 Ray, S. *et al.* α-Synuclein aggregation nucleates through liquid–liquid phase separation. *Nat. Chem.* **12**, 705-716 (2020).

2 Hardenberg, M., Horvath, A., Ambrus, V., Fuxreiter, M. & Vendruscolo, M. Widespread occurrence of the droplet state of proteins in the human proteome. *Proc. Natl. Acad. Sci. USA* **117**, 33254-33262 (2020).

3 Hardenberg, M. C. *et al.* Observation of an α-synuclein liquid droplet state and its maturation into Lewy body-like assemblies. *J. Mol. Cell Biol.* **13**, 282-294 (2021).

4 Lipiński, W. P. *et al.* Biomolecular condensates can both accelerate and suppress aggregation of α-synuclein. *Sci. Adv.* **8**, eabq6495 (2022).

5 Huang, S., Xu, B. & Liu, Y. Calcium promotes α-synuclein liquid-liquid phase separation to accelerate amyloid aggregation. *Biochem. Biophys. Res. Commun.* **603**, 13-20 (2022).

6 Piroska, L. *et al.* α-Synuclein liquid condensates fuel fibrillar α-synuclein growth. *Sci. Adv.* **9**, eadg5663 (2023).

7 Dada, S. T. *et al.* Spontaneous nucleation and fast aggregate-dependent proliferation of α-synuclein aggregates within liquid condensates at neutral pH. *Proc. Natl. Acad. Sci. USA* **120**, e2208792120 (2023).
