# Supplementary material for: Pharmacological inhibition of α-synuclein aggregation within liquid condensates
Source: Nat Commun. 2024 May 7;15:3835. doi: 10.1038/s41467-024-47585-x (PMC11076612; doi:10.1038/s41467-024-47585-x)
Supplement: Supplementary file 2 — Reporting Summary [file 41467_2024_47585_MOESM2_ESM.pdf]

nature portfolio

Corresponding author(s): Prof. Michele Vendruscolo

Last updated by author(s): 2024/03/13

Reporting Summary

Nature Portfolio wishes to improve the reproducibility of the work that we publish. This form provides structure for consistency and transparency in reporting. For further information on Nature Portfolio policies, see our Editorial Policies and the Editorial Policy Checklist.

Statistics

For all statistical analyses, confirm that the following items are present in the figure legend, table legend, main text, or Methods section.

n/a	Confirmed	
☐	☒	The exact sample size (*n*) for each experimental group/condition, given as a discrete number and unit of measurement
☐	☒	A statement on whether measurements were taken from distinct samples or whether the same sample was measured repeatedly
☐	☒	The statistical test(s) used AND whether they are one- or two-sided<br>*Only common tests should be described solely by name; describe more complex techniques in the Methods section.*
☐	☒	A description of all covariates tested
☐	☒	A description of any assumptions or corrections, such as tests of normality and adjustment for multiple comparisons
☐	☒	A full description of the statistical parameters including central tendency (e.g. means) or other basic estimates (e.g. regression coefficient) AND variation (e.g. standard deviation) or associated estimates of uncertainty (e.g. confidence intervals)
☐	☒	For null hypothesis testing, the test statistic (e.g. *F*, *t*, *r*) with confidence intervals, effect sizes, degrees of freedom and *P* value noted<br>*Give P values as exact values whenever suitable.*
☒	☐	For Bayesian analysis, information on the choice of priors and Markov chain Monte Carlo settings
☒	☐	For hierarchical and complex designs, identification of the appropriate level for tests and full reporting of outcomes
☒	☐	Estimates of effect sizes (e.g. Cohen's *d*, Pearson's *r*), indicating how they were calculated

Our web collection on statistics for biologists contains articles on many of the points above.

Software and code

Policy information about availability of computer code

Data collection	- Confocal Microscope: Leica Stellaris Will 5, LAS X STELLARIS Control Software ver.4.4.0<br>- Electron Microscopy: FEI Talos F200X G2 (S/TEM)<br>- LC-MS - Waters Vion IMS Qtof UNIFI sientific information system v1.8.2 and Waters Xevo G2-S, MassLynx 4.2 SCN 1029<br>- FTIR - OPUS software, version 7.5.18 SOFTWARE, Bruker Optics<br>- MST - Monolith NT.115, NanoTemper<br>- Nematode Tracker - GS3-U3-60QS6 M 1” Grasshopper USB 3.0 monochrome camera (Point Grey, Richmond, CA; 14 bits; 2736 × 2192 pixels) was combined with a 16 mm focal length high resolution lens f/1.8 −f/16 to image a 6–14 cm plate or a multi-well device under brightfield illumination (8” × 8” white Al side-fired backlight) (Edmund Optics Ltd.).

Data analysis	The FRAP mode from built in software of the Leica Stellaris Will 5 confocal microscope were used to obtain the fluorescence recovery. Image analysis was performed with Image J FIJI V1.53F51. The rates from aggregation assay were obtained from AmyloFit software (https://amylofit.com/amylofitmain/login/). The mass spectra were reconstructed using the MaxEnt algorithm on the MassLynx software according to the manual. MST analysis was performed using the MO. Screening Analysis Software on the Monolith NT.115 instrument. Motility assay videos were analyzed using the appropriate parameters described in the method section using in the software GUI for data analysis (Perni, M., Casford, S., Aprile, F. A., Nollen, E. A., Knowles, T. P. J., Vendruscolo, M., Dobson, C. M. Automated Behavioral Analysis of Large C. elegans Populations Using a Wide Field-of-view Tracking Platform. J. Vis. Exp. (141), e58643, doi:10.3791/58643 (2018)), Custom software written in Python (Python Software Foundation, Wilmington, Delaware, USA) was used to generate a Graphical user interface (GUI) to set up image processing and experimentalparameters, such that once tracking is completed the software captures frames stored with the camera in the.avi format.. Statistical analysis was performed using GraphPad Prism 10.0.2.

For manuscripts utilizing custom algorithms or software that are central to the research but not yet described in published literature, software must be made available to editors and reviewers. We strongly encourage code deposition in a community repository (e.g. GitHub). See the Nature Portfolio guidelines for submitting code & software for further information.

Data

Policy information about availability of data

All manuscripts must include a data availability statement. This statement should provide the following information, where applicable:

- Accession codes, unique identifiers, or web links for publicly available datasets
- A description of any restrictions on data availability
- For clinical datasets or third party data, please ensure that the statement adheres to our policy

Data availability statement was included and source data has been provided.

Research involving human participants, their data, or biological material

Policy information about studies with human participants or human data. See also policy information about sex, gender (identity/presentation), and sexual orientation and race, ethnicity and racism.

Reporting on sex and gender	No human research participants were used in this study
Reporting on race, ethnicity, or other socially relevant groupings	No human research participants were used in this study
Population characteristics	No human research participants were used in this study
Recruitment	No human research participants were used in this study
Ethics oversight	No human research participants were used in this study

Note that full information on the approval of the study protocol must also be provided in the manuscript.

Field-specific reporting

Please select the one below that is the best fit for your research. If you are not sure, read the appropriate sections before making your selection.

☒ Life sciences ☐ Behavioural & social sciences ☐ Ecological, evolutionary & environmental sciences

For a reference copy of the document with all sections, see nature.com/documents/nr-reporting-summary-flat.pdf

Life sciences study design

All studies must disclose on these points even when the disclosure is negative.

Sample size	Sample size was chosen based on the reproducibility of the data with statistically significant mean ans standard deviation. At least 3 biological samples/replicates were carried out for the analysis. Each Image captured by the microscope contained over 50 liquid droplets/condnsates and in the case of the nematode the head of the nematode. The liquid droplets were detected and quantified for size and fluorescence intensity using the image-processing pipeline in Image J (NIH). The samples were obtained and corresponding mean, standard deviations or error were calculated for their statistical significance. The P-values are reported in the respective figure legends.
Data exclusions	No data from sample were excluded during analysis. Points may have potentially been excluded by the image-processing pipeline (Image J) were a threshold function was applied to exclude the background of image and identify liquid condensates to quantify the size of individual droplets.

Replication	Experiments were repeated independently at least 3 times. Biological replicates were carried out for each measured concentration and these were performed on 3 separate occasion. All the replicates were successfully reproducible and showed similar results.
Randomization	No randomization was preformed as the study is on quantitative in vitro and in vivo experiments. Samples created were used in expriment and analysis.
Blinding	Experimentalist were not blinded in the study due to the nature of the study being an in vitro and in vivo nematode study. Data on size and fluorescence of liquid droplets were determined and obtained from Image J. C elegans experiments like FRAP data were generated from confocal software, Inclusion number was done with Image J software. Other data generated in study were generated from the droplet experiments which were not blinded.

Reporting for specific materials, systems and methods

We require information from authors about some types of materials, experimental systems and methods used in many studies. Here, indicate whether each material, system or method listed is relevant to your study. If you are not sure if a list item applies to your research, read the appropriate section before selecting a response.

Materials & experimental systems

n/a	Involved in the study
☐	☒ Antibodies
☐	☒ Eukaryotic cell lines
☒	☐ Palaeontology and archaeology
☐	☒ Animals and other organisms
☒	☐ Clinical data
☒	☐ Dual use research of concern
☒	☐ Plants

Methods

n/a	Involved in the study
☒	☐ ChIP-seq
☒	☐ Flow cytometry
☒	☐ MRI-based neuroimaging

Antibodies

Antibodies used	Alexa fluoro 488 anti his tag primary antibody (488 anti–alpha-synuclein MJFR1 (Abcam, catalog #ab195025)
Validation	This was used for western blot analysis to confirm the presence of alpha-synuclein - https://www.abcam.com/products/primary-antibodies/alexa-fluor-488-alpha-synuclein-antibody-mjfr1-ab195025.html

Eukaryotic cell lines

Policy information about cell lines and Sex and Gender in Research

Cell line source(s)	Human SH-SY5Y neuroblastoma cells (RRID:CVCL_0019)
Authentication	The cell line was authenticated using short tandem repeat (STR) analysis by the Cancer Research Uk Institute, and the cells tested negative for mycoplasma contaminations.
Mycoplasma contamination	Tested negative for mycoplasma contaminations
Commonly misidentified lines (See ICLAC register)	No commonly misidentified cell lines were used in this work

Animals and other research organisms

Policy information about studies involving animals; ARRIVE guidelines recommended for reporting animal research, and Sex and Gender in Research

Laboratory animals	C. elegans AM134 ((rmIs126[P(unc-54)Q0::YFP]), (YFP)) strain was the control strain used in this study, other strain used was, α-synuclein transgenic strain OW40 ((zgIs15 [P(unc-54)αsyn::YFP]), in which α-synuclein is expressed in the body wall muscle cells and fused to YFP. All nematodes were used in experimented were obtained as eggs.
Wild animals	No wild anaimals were used in this study
Reporting on sex	Hermaphrodite
Field-collected samples	No field collected samples
Ethics oversight	Ethical approval not required

Note that full information on the approval of the study protocol must also be provided in the manuscript.

Plants

Seed stocks	n/a
Novel plant genotypes	n/a
Authentication	n/a
